# A National Forecast and Clinical Analysis of Pediatric Acute Mastoiditis in Kazakhstan

**DOI:** 10.3390/children13020170

**Published:** 2026-01-26

**Authors:** Nazik Sabitova, Timur Shamshudinov, Assiya Kussainova, Dinara Toguzbayeva, Bolat Sadykov, Yevgeniya Rahanskaya, Laura Kassym

**Affiliations:** 1 Higher School of Public School, Kazakhstan Medical University, Almaty 050060, Kazakhstan; 2 Medical and Diagnostic Center of Otorhinolaryngology, Almaty 050000, Kazakhstan; 3 Department of General Medical Practice with a Course of Evidence-Based Medicine, Astana Medical University, Astana 010000, Kazakhstan; 4 Department of Otorhinolaryngology, NEI Kazakh-Russian Medical University, Almaty 050000, Kazakhstan; 5 Center of Pediatric Otorhinolaryngology, General Hospital #5, Almaty 050000, Kazakhstan; 6 Department of Traumatology and Pediatric Surgery, Semey Medical University, Semey 071400, Kazakhstan

**Keywords:** children, pediatricians, otolaryngology, acute mastoiditis, otitis media, Kazakhstan

## Abstract

**Highlights:**

**What are the main findings?**

• Pediatric acute mastoiditis reveals a growing mismatch between healthcare demand and workforce and between healthcare demand and inpatient capacity in Kazakhstan.
• Declining numbers of pediatricians and ENT beds coexist with stable ENT workforce density and a rising volume of ENT surgeries.

**What are the implications of the main findings?**

• Acute mastoiditis can serve as a sentinel condition for assessing systemic constraints in pediatric otorhinolaryngology.
• Integrated health policies addressing workforce planning, inpatient capacity, and referral pathways are essential to prevent further growth of complicated pediatric ENT conditions.

**Abstract:**

**Background:** Ongoing healthcare and medical education reforms in Kazakhstan have been accompanied by persistent workforce shortages and reduced inpatient capacity in pediatric care. Therefore, this study aimed to assess and forecast selected healthcare system indicators using acute mastoiditis (AM) as a sentinel condition while also describing its clinical and epidemiological characteristics. **Materials and Methods:** This study combined an analysis of national healthcare and demographic statistics in Kazakhstan from 1998 to 2024 with a retrospective review of pediatric AM patients treated at a tertiary referral center. Long-term trends in healthcare resources were assessed, and future needs were projected via average annual percentage change (AAPC) and time series forecasting methods. Clinical, laboratory, and radiological data were extracted from medical records. Statistical analyses were performed via SPSS version 24.0 (IBM Corp., Armonk, NY, USA). **Results:** From 1998 to 2024, the number of pediatricians and ENT hospital beds declined, whereas the density of ENT physicians remained relatively stable, and the proportion of ENT surgical procedures increased. Projections to 2030 suggest continued constraints in pediatric and ENT workforce capacity and further reductions in inpatient beds despite sustained growth in surgical demand. Among 95 pediatric AM cases, complications, most commonly subperiosteal abscess and zygomatic abscess, were identified in 40% of patients. **Conclusions:** AM may be considered a contextual indicator of pressures within specialized pediatric ENT services rather than a direct measure of healthcare system performance. These findings highlight the need for further studies to validate these observations and better inform healthcare planning.

## 1. Introduction

Following the dissolution of the Soviet Union, post-Soviet countries implemented a series of healthcare and medical education reforms that resulted in substantial structural changes in the organization and delivery of medical services [1]. Evaluations of these reforms indicate that the transition was accompanied by considerable challenges related to workforce distribution and adaptation to new models of healthcare provision in Armenia, Belarus, Kazakhstan, Kyrgyzstan, Tajikistan, and other countries in the region [2]. In Kazakhstan, a major reform of higher medical education in 2007 led to the discontinuation of pediatric faculties and the reorientation of pediatric training toward a general practitioner-based model [3]. These changes contributed to a long-term shortage of pediatric specialists and reduced access to specialized care for children. In response to the growing workforce gap, Kazakhstan initiated the gradual reinstatement of pediatric training programs from 2017 to 2018 [4]. Nevertheless, despite the resumption of education, workforce capacity remains limited, particularly in highly specialized fields such as pediatric otorhinolaryngology (ear, nose, and throat, ENT) [5]. An additional constraining factor is the ongoing optimization of inpatient care, including reductions in hospital bed capacity and shorter lengths of stay [6], which may further limit the timely diagnosis and management of severe and complicated ENT conditions in children.

In this context, acute mastoiditis (AM) represents a useful clinical model for assessing the impact of systemic constraints within pediatric otorhinolaryngology. AM is a severe bacterial infection of the mastoid process that most commonly develops as a complication of acute otitis media and is characterized by inflammation and destruction of mastoid air cells, with a risk of infection spreading to adjacent anatomical structures [7,8]. Although the incidence of AM has declined in high-income countries, it remains a clinically important condition, particularly in pediatric practice [9]. According to published data, the global incidence of mastoiditis ranges from 1.2 to 1.6 cases per 100,000 people per year, with the highest burden observed among young children [10,11]. The increased vulnerability of this age group is attributed to immune system immaturity [12], as well as the increasing prevalence of antimicrobial resistance [13,14]. The reported rates of intracranial complications associated with AM vary from 5% to 29% and include sigmoid sinus thrombosis, epidural and subdural abscesses, suppurative labyrinthitis, and facial nerve paralysis [15].

Despite its clinical importance, systematically collected national-level epidemiological data on the incidence of acute otitis media and its complications, including AM, are largely lacking in Kazakhstan. The available evidence remains fragmented and is typically confined to reports from individual hospitals or referral centers, as not all healthcare facilities have the capacity to provide specialized ENT care. Another restriction is the absence of an updated national clinical guideline [16] that provides clearly defined management strategies for complicated forms of otitis media and mastoiditis, which may contribute to variability in clinical decision-making and delays in timely referral to specialized institutions.

Accordingly, the aim of the present study was to assess and forecast healthcare system indicators in Kazakhstan via AM as a model condition while simultaneously examining the clinical and epidemiological characteristics of AM.

## 2. Materials and Methods

### 2.1. Study Design

This study was conducted in several two stages. This integrated, two-part design was implemented to address the management of pediatric AM from a systems-based perspective. First, national healthcare statistics were analyzed to forecast future population needs in Kazakhstan. This macrolevel analysis aimed to identify systemic trends, potential resource gaps, and the epidemiological context of pediatric ENT care at the national level, providing a backdrop for clinical findings. Second, a retrospective review of medical records from a large Kazakhstani tertiary referral center in Almaty was conducted to characterize the contemporary clinical presentation, management pathways, and outcomes of AM; data from the tertiary referral center in the capital city of Astana were not available for analysis. This single-center, in-depth clinical analysis aimed to provide detailed real-world insights into disease presentation and current management practices that are not routinely captured in national healthcare statistics.

### 2.2. Evaluation of Nationwide Demographic and Healthcare Data

The official healthcare statistics were obtained from the statistical yearbooks “Health of the Population of the Republic of Kazakhstan and Activities of Healthcare Organizations”, compiled by the Ministry of Healthcare of the Republic of Kazakhstan and publicly available on the website of the Salidat Kairbekova National Scientific Center for Healthcare Development [17]. The retrieved data included (i) the number of pediatricians per 10,000 people, (ii) the number of ENT doctors per 10,000 people, (iii) the availability of ENT hospital beds per 10,000 people, and (iv) the share of ENT surgeries within the overall volume of surgical procedures for ear surgeries. The healthcare statistics used in this study spanned the period from 1998 to 2024, which represents the full range of publicly accessible national data.

### 2.3. Retrospective Analysis of Pediatric AM Patients

A descriptive analysis was performed at a major referral center in Almaty, Kazakhstan (ENT Department, City Clinical Hospital No. 5). Medical records were extracted from the computerized hospital database and included all children admitted with AM between 1 September 2022, and 29 February 2024. Children were admitted to the center with a diagnosis of AM, which was confirmed by an otolaryngologist. The diagnosis was based on the presence of AOM, fever, otalgia, and retroauricular swelling and erythema. Medical records were reviewed for demographic data (age and sex), clinical presentations (fever, retroauricular erythema and swelling, otorrhea, pain on palpation, and fluctuance), laboratory parameters (hemoglobin, leukocyte count, platelet count, and ESR), and radiological findings (temporal bone computed tomography). Additionally, the type of complication (if present) and hospitalization details (total hospital stay and length of postoperative hospitalization in cases requiring surgery) were recorded. The inclusion criteria were as follows: (a) age 0–18 years and (b) the presence of clinical and radiological signs of AM [18]. Radiological confirmation via temporal bone computed tomography (CT) was required for all included patients. CT findings were used both to confirm the diagnosis of AM and to determine disease severity. Accordingly, incipient mastoiditis was classified as a moderate form of AM, whereas coalescent mastoiditis was defined as severe [19]. As severity classification relies exclusively on radiological criteria, the observed distribution of moderate and severe cases reflects radiologically confirmed disease.

### 2.4. Ethics

The study was approved by the Ethics Committee of Kazakhstan’s Medical University “Kazakhstani School of Public Health” (Approval No. 8, dated 6 May 2025). Parental informed consent was obtained for the use of retrospective medical records in accordance with institutional and national ethical requirements. All procedures were conducted in compliance with the principles of the Declaration of Helsinki.

### 2.5. Statistical Analysis

Healthcare statistics covering the period from 1998 to 2024 were collated in Microsoft Excel© for subsequent analysis. Historical trends were quantified via the average annual percentage change (AAPC), which summarizes the mean yearly change over the study period and was calculated from log-linear regression models fitted to the time series data. AAPC estimates are presented with corresponding 95% confidence intervals (CIs) to reflect statistical uncertainty. The forecasting of epidemiological trends through 2030 was performed via time series analysis in SPSS. The Expert Modeler function was applied to automatically identify the most appropriate forecasting model on the basis of goodness-of-fit statistics and residual diagnostics. Depending on the characteristics of the data, autoregressive integrated moving average (ARIMA) models were selected for series exhibiting autocorrelation and potential nonstationarity, whereas Holt’s exponential smoothing models were used for series demonstrating a consistent linear trend without pronounced seasonality. Model adequacy was assessed via standard fit criteria and examination of residuals.

Projections are reported as point estimates with corresponding 95% confidence intervals. The width of the 95% confidence intervals was used to reflect uncertainty in the projections, with wider intervals indicating lower precision of long-term forecasts. Projected point estimates were therefore interpreted as indicative trends rather than exact future values, and all results are reported with corresponding confidence intervals to ensure transparent uncertainty assessment. Visualizations were generated to depict both observed and projected values.

Continuous variables are presented as medians with interquartile ranges (IQRs). The normality of continuous data was assessed via the Shapiro–Wilk test. As the data were nonnormally distributed, between-group comparisons for continuous measures were performed via the Mann–Whitney U test. Categorical variables are presented as counts and percentages and were compared between groups via the chi-square test. All the statistical analyses were conducted via SPSS for Windows, version 24.0 (IBM Corp., Armonk, NY, USA). A two-sided *p* value < 0.05 was considered statistically significant.

## 3. Results

### 3.1. Longitudinal Trends and Forecasted Availability of Pediatric and ENT Healthcare Resources

Table S1 presents longitudinal data on pediatric and otorhinolaryngology healthcare resources in Kazakhstan from 1998 to 2024 (Table S1). The number of pediatricians per 10,000 population showed considerable variability over the study period. After a peak of 5.5 in 2002, a general declining trend was observed, with values falling from 4.0 in 2011 to 2.5 in 2024. The AAPC was −2.58% (95% CI: −3.16, −2.00; *p* < 0.001), indicating a statistically significant annual decrease over the full period. The number of ENT doctors remained relatively low and stable, fluctuating between 0.1 and 1.0 per 10,000 people. From 2011 to 2024, the rate stabilized at 0.6–0.7. The AAPC of 1.05% was not statistically significant (95% CI: −1.28, 3.44; *p* = 0.183). The availability of ENT hospital beds per 10,000 people exhibited a clear downward trajectory, declining from 1.0 in 1998 to 0.5 by 2024. The AAPC was −3.62% (95% CI: −4.19, −3.05; *p* < 0.001), reflecting a significant and steady reduction in bed capacity. The share of ENT surgeries among all surgical procedures demonstrated a consistent upward trend, rising from 0.4% in 1998 to 1.3% in 2024, with notable increases in 2008 and 2018–2024. The AAPC was 4.29% (95% CI: 3.59, 5.00; *p* < 0.001), indicating a significant annual increase in the relative volume of ENT surgeries.

Table 1 presents the projected values for key pediatric and otorhinolaryngology healthcare indicators in Kazakhstan for the period 2027–2035, which are based on time series modeling of historical data. The projected number of pediatricians per 10,000 population is expected to remain stable at approximately 2.6–2.7 throughout the forecast period, with widening 95% confidence intervals over time. The ARIMA (4,1,0) model indicates a statistically significant declining trend in the historical data (*p* < 0.001). The number of ENT doctors is projected to remain unchanged at 0.6 per 10,000 population across all forecasted years, as supported by a significant ARIMA (0,0,1) model (*p* < 0.001). In contrast, the availability of ENT hospital beds per 10,000 people is projected to decrease gradually from 0.5 in 2027 to 0.3 by 2035. This downward trend, modeled via ARIMA (0,1,0), was marginally nonsignificant in the historical series (*p* = 0.057). The share of ENT surgeries within the total volume of surgical procedures is projected to increase steadily from 1.3% in 2027 to 1.5% by 2035. The Holt model did not demonstrate a statistically significant historical trend (*p* = 0.468), although a gradual increase is forecasted.

**Figure 1:**
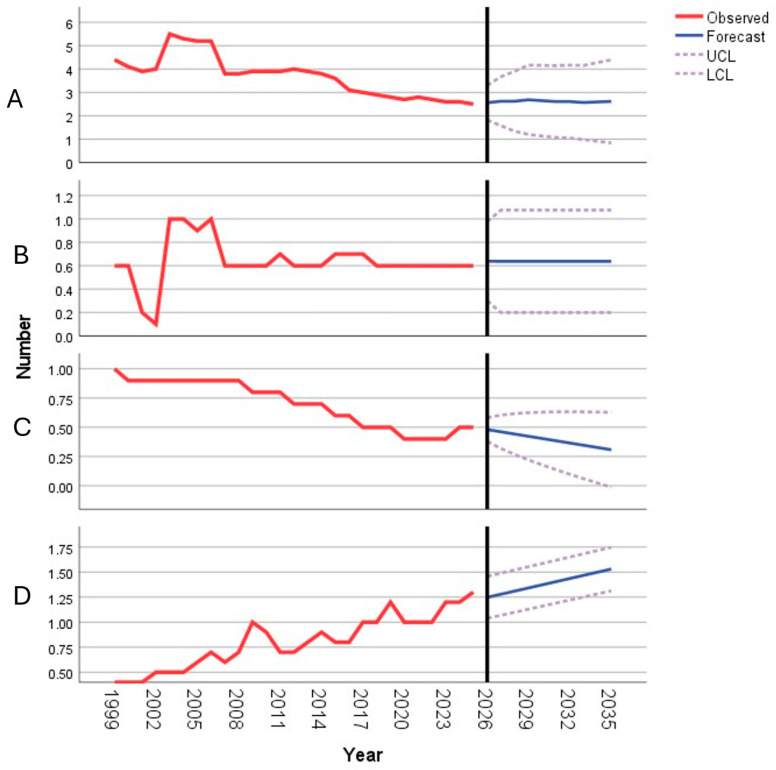
Forecasted availability of pediatricians (**A**), ENT specialists (**B**), ENT hospital beds (**C**), and ENT surgeries (**D**) in Kazakhstan (2026–2030). (UCL—Upper Confidence Limit; LCL—Lower Confidence Limit).

Figure 1 illustrates the forecasted availability of key pediatric and otorhinolaryngology healthcare indicators in Kazakhstan for the period 2026–2030 and complements the numerical projections presented in Table 1. The number of pediatricians per 10,000 population is projected to remain largely stable over the forecast horizon (Figure 1A). Similarly, the number of ENT specialists is expected to show minimal change, maintaining a constant level throughout the period (Figure 1B). In contrast, the availability of ENT hospital beds demonstrated a slight downward trend (Figure 1C). Conversely, the share of ENT surgeries within the overall volume of surgical procedures is projected to increase gradually over time (Figure 1D), indicating a growing surgical workload despite a stable workforce capacity.

### 3.2. Comparison of the Clinical Characteristics of Noncomplicated and Complicated Acute Mastoiditis Patients

To contextualize these national healthcare resource trends within patient outcomes, a clinical case series of pediatric acute mastoiditis patients was analyzed, and the results are presented below. Over an 18-month retrospective observation period, 95 patients with AM who met the inclusion criteria were identified. The mean age of the patients was 74.92 months, with a range from 4 to 193 months. Of these, 58.9% (n = 56) were male, and the vast majority resided in urban areas. All 95 patients presented with fever, swelling, otorrhea, and pain on palpation. Retroauricular erythema was observed in 94.7% of the patients (n = 90), whereas fluctuations were detected in 43.2% of the patients (n = 46). In 42.1% of the patients (n = 40), AM affected the left side; right-sided involvement was observed in 35.8% (n = 34), and bilateral AM was identified in 22.1% of the patients (n = 22). Eighty-nine patients were admitted to the hospital with moderate disease severity, while six were classified as severely ill. All patients had adenoiditis as a comorbidity.

Complications developed in 38 patients with AM (40%). Among the observed complications, subperiosteal abscess was identified as one of the most common manifestations (n = 20), followed by zygomatic abscess (n = 13). Isolated cases of sigmoid sinus thrombosis (n = 1), facial paresis (n = 1), a combination of facial paresis and meningitis (n = 1), subperiosteal abscess with sigmoid sinus thrombosis (n = 1), and subperiosteal abscess with facial paresis (n = 1) were also identified.

Table 2 presents the differences between patient subgroups with noncomplicated AM and those with complicated AM. The median age and sex ratio did not differ significantly between the groups (*p* = 0.882 and *p* = 0.055, respectively). Urban residency was prevalent in both groups, with no significant difference (*p* = 0.449). Severe cases were observed exclusively in the complicated AM group (15.8%, *p* = 0.002), as was rapid disease progression within 7 days (7.9%, *p* = 0.031). Retroauricular erythema was common in both groups, although it was more common in complicated cases (100% vs. 91.2%, *p* = 0.061), whereas retroauricular fluctuance was significantly different (76.3% vs. 21.1%, *p* = 0.001). Radiologically, temporal bone destruction on CT was markedly more prevalent in the complicated group (84.2% vs. 3.5%, *p* = 0.001). Laboratory parameters revealed a significantly greater leukocyte count (*p* = 0.001) and ESR (*p* = 0.019) in complicated patients than in uncomplicated patients, whereas hemoglobin and platelet counts were not significantly different. Patients with complicated AM had longer total hospital stays (median of 5 vs. 4 days, *p* = 0.001) and longer postoperative hospitalization periods (median of 4 vs. 3 days, *p* = 0.001).

Table 3 shows the distribution of surgical interventions among patients diagnosed with noncomplicated and complicated AM. The most frequently performed procedure among patients with noncomplicated AM (n = 57) was myringostomy, reported in 96.5% of patients (55/57), whereas it was performed in only 15.8% (6/38) of patients with complicated AM (n = 38). Conversely, atticoantrotomy was predominant in the complicated AM group, with a frequency of 60.5% (23/38), compared with only 1.8% (1/57) in the noncomplicated group. A combination of myringostomy on the contralateral side with atticoantrotomy was performed in 13.2% (5/38) of the complicated patients and in 1.8% (1/57) of the noncomplicated patients. Mastoidectomy was exclusively reported in the complicated group, accounting for 10.5% (4/38) of the cases, and was not performed in any of the noncomplicated patients.

## 4. Discussion

### 4.1. Forecasted Healthcare Demand and Workforce Capacity Challenges

A negative AAPC indicates a steady decrease in pediatrician availability in Kazakhstan, which is consistent with the long-term consequences of earlier healthcare system reforms. Analytical reports by the World Health Organization (WHO) indicate that such workforce shifts may increase the risk of delayed detection of severe conditions in children and contribute to a greater proportion of complicated cases requiring inpatient care and surgical intervention [3]. In this context, assessments of pediatric care quality in hospitals in Kyrgyzstan and Tajikistan have shown that shortages of pediatricians substantially limit the capacity of healthcare facilities to deliver high-quality medical services [20]. Moreover, Sakai, R. et al. (2016) demonstrated that higher pediatric workforce density is associated with lower mortality rates among children under five years of age [21].

In light of this, a persistently rising percentage of ENT surgical procedures and a comparatively stable but low density of otorhinolaryngologists point to the establishment of a mismatch between the supply and demand of specialized treatment. A similar “supply–demand gap” has been described in the international literature on workforce planning in otorhinolaryngology, where modeling studies demonstrate the risk of misalignment between population needs and specialist availability when service demand increases more rapidly than workforce capacity [22,23]. In Kazakhstan, this imbalance may be further exacerbated by the continued growth of the pediatric population [24], leading to increased utilization of ENT services and an increasing need for surgical care.

In addition, reductions in inpatient bed capacity occurring alongside increasing surgical volumes and continued growth of the pediatric population may constrain the ability of healthcare systems to adequately manage severe and complicated conditions, including AM. International experience indicates that the optimization of inpatient care without concurrent strengthening of outpatient and specialized services is associated with extended hospital stays, increased pressure on surgical departments, and a heightened risk of delays in the delivery of timely care for acute pediatric conditions [25].

### 4.2. Incidence and Complication Profile of Pediatric AM

Over an 18-month retrospective observation period at a referral center in Almaty, Kazakhstan, 95 pediatric patients were diagnosed with AM, indicating an exceptionally high case burden over a relatively short timeframe. This finding is particularly notable compared with international reports [26,27,28]. For example, a review of electronic medical records from one of the largest pediatric hospitals in the United States identified only 129 AM cases over an eight-year period (2011–2019) [26], whereas Karaaslan et al. (2023) reported only 28 hospitalizations for AM over seven years at a municipal hospital in Istanbul, Turkey [27]. In contrast, data from the Israeli national registry revealed an incidence of 7.78 cases per 100,000 child-years, exceeding rates reported in other Western countries [28].

The 40% AM complication rate reported in this study is higher than that reported in several investigations worldwide [29]. This difference is likely multifactorial and may partly reflect referral bias, as the clinical cohort was drawn from a tertiary referral center that predominantly manages more severe and advanced cases. In addition, diagnostic thresholds and case definitions, including the routine use of CT, may have contributed to a higher detection rate of bony involvement and complications than studies using more conservative diagnostic approaches. Furthermore, the high proportion of complicated cases may reflect delayed outpatient diagnosis and late hospital presentation, as indicated by the frequent presence of retroauricular erythema and fluctuation and radiological evidence of bone destruction in 84.2% of patients.

A comparison with published data revealed substantial variability in the reported complication rates of AM, ranging from 1.9% to 35% [29]. For example, Guillén-Lozada et al. (2022) reported that 109 of 412 children with AM (26.5%) developed severe intracranial complications, which significantly influenced treatment strategies and the length of hospital stay [30]. In contrast, another study reported complications in only 9.5% of 147 AM patients and reported a significant inverse association between retroauricular erythema and the likelihood of complications [29].

In the present study, elevated leukocyte counts and ESR emerged as the most informative laboratory markers in complicated AM, underscoring the importance of close laboratory monitoring to prevent adverse outcomes. These findings are supported by a study from Lisbon involving 135 children with AM, which demonstrated a significant association between increased leukocyte counts, elevated C-reactive protein levels, and the development of complications [31]. Moreover, several authors emphasize the ESR as a particularly valuable parameter for guiding decisions regarding both antimicrobial therapy and the need for surgical intervention [32].

Myringotomy was the predominant surgical intervention in uncomplicated AMs (96.5%), whereas atticoantrotomy was more common in complicated cases (60.5%), reflecting disease severity. This pattern aligns with published data, as myringotomy is a minimally invasive procedure used for middle ear drainage, whereas atticoantrotomy and, less frequently, mastoidectomy (10.5%) are reserved for advanced cases with bony involvement [33,34]. Some authors, including Mierzwiński et al. (2019), have suggested early mastoidectomy in patients unresponsive to 48 h of intravenous antibiotic therapy to reduce the risk of recurrence [35].

### 4.3. System-Level Preventive Strategies and Policy Implications

Kazakhstan’s healthcare system continues to face structural challenges, including workforce shortages, medical staff migration, and pronounced urban–rural disparities in resource distribution [36]. Additional constraints arise from the high level of infrastructure depreciation and the misalignment of healthcare tariffs with actual service costs and inflationary pressures [37]. Against this background, the clinical profile of pediatric AM observed in the present study—characterized by a high proportion of complicated cases—may reflect the downstream effects of workforce shortages, uneven access to timely care, and limitations at the primary and secondary care levels. However, preventing complicated forms of pediatric diseases cannot be viewed solely through the lens of increasing physician numbers and instead requires coordinated, system-level interventions.

One key priority is maintaining high immunization coverage, as declining vaccination rates against vaccine-preventable infections have been associated with an increased incidence of otitis media and related conditions [38]. For example, Daniel et al. (2013) demonstrated that routine pneumococcal vaccination was associated with a significant reduction in invasive ENT infections and pediatric hospitalizations [39]. Equally important is improving parental health literacy and awareness, given the persistent trends of vaccine refusal and self-medication among caregivers in the country [40].

Systematic strengthening of healthcare workforce capacity, including continuous professional development supported by remote education and telemedicine tools [41], as well as sustained interdisciplinary collaboration between clinicians and microbiological laboratories, may improve early diagnosis and optimize treatment strategies. At the same time, the absence of a unified, evidence-based clinical management pathway for complicated pediatric infections highlights the need for the development of standardized approaches aligned with international guidelines and adapted to local healthcare system capacities [16]. According to the Organization for Economic Co-operation and Development (OECD), strengthening research capacity in primary health care and expanding the clinical and decision-making roles of general practitioners represent some of the most effective approaches to reducing pressure on inpatient and specialized care services [42]. Finally, revising remuneration policies, implementing targeted retention and incentive strategies for physicians in both urban and rural settings, developing dedicated investment programs for healthcare infrastructure modernization are essential to ensure the long-term sustainability and resilience of the national healthcare system.

### 4.4. Study Limitations

This study has several limitations that should be acknowledged. First, the retrospective design based on medical record review may be subject to information bias, as the completeness and quality of documentation could not be fully controlled. Although all available clinical, laboratory, and radiological data were extracted, variations in record-keeping practices may have influenced the consistency of certain variables.

Second, the clinical analysis was conducted at a single tertiary referral center, which may limit the generalizability of the findings. Referral bias may have contributed to the higher observed incidence and complication rates of AM, and the results may not fully represent national epidemiological trends. Moreover, reliance on AM as a sole indicator may limit the robustness of conclusions regarding healthcare system constraints, which are ideally evaluated via multiple complementary conditions.

Third, the absence of a centralized national registry for acute otitis media and its complications in Kazakhstan restricts the ability to validate findings against population-based data.

## 5. Conclusions

AM should not be regarded as a direct measure of healthcare system performance but may serve as a contextual indicator reflecting pressures within specific components of the healthcare system, particularly specialized and inpatient services. The observed trends suggest that system-level constraints may influence clinical presentation and management; however, broader validation across multiple centers, levels of care, and clinical conditions is needed to confirm the generalizability of these findings. Overall, this study highlights the value of integrating health system indicators with clinical data to better characterize emerging challenges in pediatric care and underscores the need for future studies incorporating additional sentinel conditions to assess healthcare system capacity more comprehensively and support evidence-based planning and policy development.

## Figures and Tables

**Table 1:** The projected numbers of pediatricians, ENT doctors, ENT hospital beds, and shares of ear-related surgeries in Kazakhstan for the years 2026 and 2030, accompanied by 95% confidence intervals.

Year	Number of Pediatricians (per 10,000 Population) Number (95% CI)	Number of ENT Doctors (per 10,000 Population) Number (95% CI)	Availability of ENT Hospital Beds per 10,000 Population Number (95% CI)	Share of ENT Surgeries within the Overall Volume of Surgical Procedures Ear Surgeries Number (95% CI)
2027	2.6 (1.6; 3.7)	0.6 (0.2; 1.1)	0.5 (0.3; 0.6)	1.3 (1.1; 1.5)
2028	2.6 (1.3; 3.9)	0.6 (0.2; 1.1)	0.4 (0.3; 0.6)	1.3 (1.1; 1.5)
2029	2.7 (1.2; 4.2)	0.6 (0.2; 1.1)	0.4 (0.2; 0.6)	1.3 (1.1; 1.6)
2030	2.6 (1.1; 4.2)	0.6 (0.2; 1.1)	0.4 (0.2; 0.6)	1.4 (1.2; 1.6)
2031	2.6 (1.1; 4.2)	0.6 (0.2; 1.1)	0.4 (0.1; 0.6)	1.4 (1.2; 1.6)
2032	2.6 (1.0; 4.2)	0.6 (0.2; 1.1)	0.4 (0.1; 0.6)	1.4 (1.2; 1.7)
2033	2.6 (1.0; 4.2)	0.6 (0.2; 1.1)	0.3 (0.1; 0.6)	1.5 (1.3; 1.7)
2034	2.6 (0.9; 4.3)	0.6 (0.2; 1.1)	0.3 (0.0; 0.6)	1.5 (1.3; 1.7)
2035	2.6 (0.8; 4.4)	0.6 (0.2; 1.1)	0.3 (0.0; 0.6)	1.5 (1.3; 1.7)
Model parameters	ARIMA (4, 1, 0), *p* < 0.001	ARIMA (0, 0, 1), *p* < 0.001	ARIMA (0, 1, 0), *p* = 0.057	Holt *p* = 0.468

**Table 2:** Comparative characteristics of patients with noncomplicated and complicated AM.

	Noncomplicated AM (n = 57)	Complicated AM (n = 38)	*p* Value
Age (Me (range)) (months)	63 (33–106)	70 (34–90)	0.882
Male/Female Ratio	1.66	1.24	0.055
Urban residency	51 (89.5%)	32 (84.2%)	0.449
Clinical presentation			
Severe cases	0 (0%)	6 (15.8%)	0.002
Rapid progress (<7 days)	0 (0.0%)	3(7.9%)	0.031
Retroauricular erythema	52 (91.2%)	38 (100.0%)	0.061
Retroauricular fluctuance	12 (21.1%)	29 (76.3%)	0.001
CT findings			
Destruction of temporal bone	2 (3.5%)	32 (84.2%)	0.001
Laboratory parameters			
Hemoglobin (Me (range)) (g/L)	113 (104–121)	115 (104–121)	0.948
Leucocyte count (Me (range)) (×10^9^/L)	10 (9–13)	15 (11–17)	0.001
Platelet count (Me (range)) (×10^9^/L)	344 (306–458)	346 (252–470)	0.477
ESR (Me (range)) (mm/h)	37 (27–48)	42 (38–49)	0.019
Hospitalization details			
Total hospital stay (days)	4 (4–4)	5 (5–5)	0.001
Length of postoperative hospitalization (in case of surgery) (days)	3 (3–3)	4 (4–4)	0.001

**Table 3:** Types of surgeries performed in patients with noncomplicated and complicated AM.

Type of Intervention	Noncomplicated AM (n = 57)	Complicated AM (n = 38)
Myringostomy	55 (96.5%)	6 (15.8%)
Atticoantrotomy	1 (1.8%)	23 (60.5%)
Myringostomy on the other side in combination with atticoantrotomy	1 (1.8%)	5 (13.2%)
Mastoidectomy	0 (0.0%)	4 (10.5%)

## Data Availability

The original data presented in the study are openly available at GitHub at https://github.com/laurakassym-a11y/Manuscript-Sabitova-Dataset (accessed on 1 December 2025).

## Supplementary Materials

The following supporting information can be downloaded at: https://www.mdpi.com/article/10.3390/children13020170/s1. Table S1: The numbers of pediatricians, ENT doctors, ENT hospital beds, and shares of ear-related surgeries in Kazakhstan (1998–2024).

## Abbreviations

The following abbreviations are used in this manuscript:

AM	Acute mastoiditis
ESR	Erythrocyte sedimentation rate
CRP	C-reactive protein
CT	Computed tomography
MRI	Magnetic resonance imaging
RK	Republic of Kazakhstan
WHO	World Health Organization
AAPC	Average annual percentage change
ENT	Ear, nose, and throat
ARIMA	Autoregressive integrated moving average
CIs	Confidence intervals
IQRs	Interquartile ranges
