# Supplementary material for: A National Forecast and Clinical Analysis of Pediatric Acute Mastoiditis in Kazakhstan
Source: Children (Basel). 2026 Jan 26;13(2):170. doi: 10.3390/children13020170 (PMC12939994; doi:10.3390/children13020170)
Supplement: Supplementary file 1 [file children-13-00170-s001.zip › children-4092768-supplementary.pdf]

Supplementary Table S1. The numbers of pediatricians, ENT doctors, ENT hospital beds, and share of ear-related surgeries in Kazakhstan (1998-2024)

**Parameter**	**1998**	**1999**	**2000**	**2001**	**2002**	**2003**	**2004**	**2005**	**2006**	**2007**	**2008**	**2009**	**2010**	**2011**	**2012**	**2013**	**2014**	**2015**	**2016**	**2017**	**2018**	**2019**	**2020**	**2021**	**2022**	**2023**	**2024**	**AAPC* (95%CI, p Value**
Number of pediatricians (per 10 000 population)	4,4	4,1	3,9	4	5,5	5,3	5,2	5,2	3,8	3,8	3,9	3,9	3,9	4	3,9	3,8	3,6	3,1	3	2,9	2,8	2,7	2,8	2,7	2,6	2,6	2,5	-2.58% (-3.16,-2.00, p<0.001)
Number of ENT doctors (per 10 000 population)	0,6	0,6	0,2	0,1	1	1	0,9	1	0,6	0,6	0,6	0,6	0,7	0,6	0,6	0,6	0,7	0,7	0,7	0,6	0,6	0,6	0,6	0,6	0,6	0,6	0,6	1.05% (-1.28,3.44, p=0.183
Availability of ENT hospital beds per 10000 population	1	0,9	0,9	0,9	0,9	0,9	0,9	0,9	0,9	0,9	0,8	0,8	0,8	0,7	0,7	0,7	0,6	0,6	0,5	0,5	0,5	0,4	0,4	0,4	0,4	0,5	0,5	-3.62% (-4.19,-3.05, p<0.001)
Share of ENT surgeries within the overall volume of surgical procedures ear surgeries	0,4	0,4	0,4	0,5	0,5	0,5	0,6	0,7	0,6	0,7	1	0,9	0,7	0,7	0,8	0,9	0,8	0,8	1	1	1,2	1	1	1	1,2	1,2	1,3	4.29% (3.59.5.00, p<0.001)
